# Research frontiers and hotspots in bacterial biofilm wound therapy: bibliometric and visual analysis for 2012–2022

**DOI:** 10.1097/MS9.0000000000001321

**Published:** 2023-09-14

**Authors:** Rong Liu, Linjun Zhai, Shengya Feng, Rong Gao, Jie Zheng

**Affiliations:** Department of Nursing, Shanxi Medical University, Taiyuan, Shanxi Province, China

**Keywords:** microbiology, treatment, web of science, wound bacterial biofilm, wound healing

## Abstract

**Background:**

Bacterial biofilms, which can protect bacteria from host immune response and drug attack, are an important factor in the difficult healing of chronic wound infection, which has become a major problem in medical development. This paper aimed to analyze literature related to bacterial biofilm wound treatment published between 2012 and 2022 using bibliometric and visual analysis.

**Methods:**

Publications related to bacterial biofilm wound treatment from 2012 to 2022 were selected from the Web of Science Core Collection. Microsoft Excel 2021, bibliometrics, CiteSpace6.1, and VOSviewer1.6.18 were used to extract and analyze data.

**Results:**

A total of 940 articles were published between 2012 and 2022, with the United States being the leading country (with 302 papers, 32.13%) and the University of Copenhagen in Denmark being the leading institution (with 26 published articles) in the field. Steven L Percival, a British academic, published the most articles (14). In the field of bacterial biofilm wound treatment, keywords suggested that the research gradually transitioned from lower limb venous ulcer, negative pressure-assisted healing to chronic wound, in-vitro bacterial biological model research, and then to the development of more microscopic and more advanced technologies such as antibacterial activity and nanomaterials. “Nanoparticles”, “inhibition/antibacterial”, “delivery”, “gold nanoparticles”, “hydrogel”, “wound healing”, etc., may become new research hotspots in this field.

**Conclusion:**

There is a lack of specific and effective treatment methods for diagnosing and treating bacterial biofilms in wounds. Through the development of multidisciplinary cooperation, early diagnosis and treatment of bacterial biofilms in wounds can be achieved. These data may provide a useful reference for scholars studying more effective bacterial biofilm wound treatment.

## Introduction

Highlights

- Wound bacterial biofilms represent a particularly challenging problem in modern medicine, it has attracted the attention of many scholars.
- This research explore the current research status of bacterial biofilm wound treatment employing bibliometric analysis methods, it can help researchers and clinical medical staff to better understand the research hotspots and development trends.
- Multiple bibliometrics analysis tools were used for comprehensive analysis.

Wound infections by pathogens or biofilms, characterized by invasion by proliferating microorganisms to a level that invokes a local and/or systemic response in the host, can lead to chronic wound healing and slow tissue repair, hindering timely resolution^1^. Bacteria that form biofilms and participate in wound infections are particularly difficult to treat because these bacteria form robust biofilms that embed bacterial cells in a self-produced polymer matrix that is protected from host immune responses and antibiotics, providing the community with many advantages such as metabolic cooperation, passive resistance, and horizontal gene transfer^2^. They can be up to 1000 times more resistant to antimicrobials, disinfectants, and the host immune system than bacteria^3^. Recent data suggest that biofilm-associated infections account for half a million deaths annually, resulting in severe economic losses estimated at about $94 billion per year^4^. Current care measures for wound biofilms include frequent removal of biofilms through conventional treatment, including debridement, local and systemic antibiotics, preservatives, and wound dressings^5^. There is a lack of effective treatment for wound biofilm infection, and alternative treatment options are urgently needed^6^. However, there is still a lack of effective diagnosis and intervention measures for wound bacterial biofilm. Bibliometrics, sometimes referred to as scientometrics, is a mathematical and statistical method of quantitative analysis based on changes in citations, authors, keywords, number of papers, and other measures^7^. This study aimed to explore the current research status of bacterial biofilm wound treatment employing bibliometric analysis and traditional review methods, clarifying research trends and hotspots, and providing references for researchers.

## Methods

### Data source and search strategies

**Figure 1:**
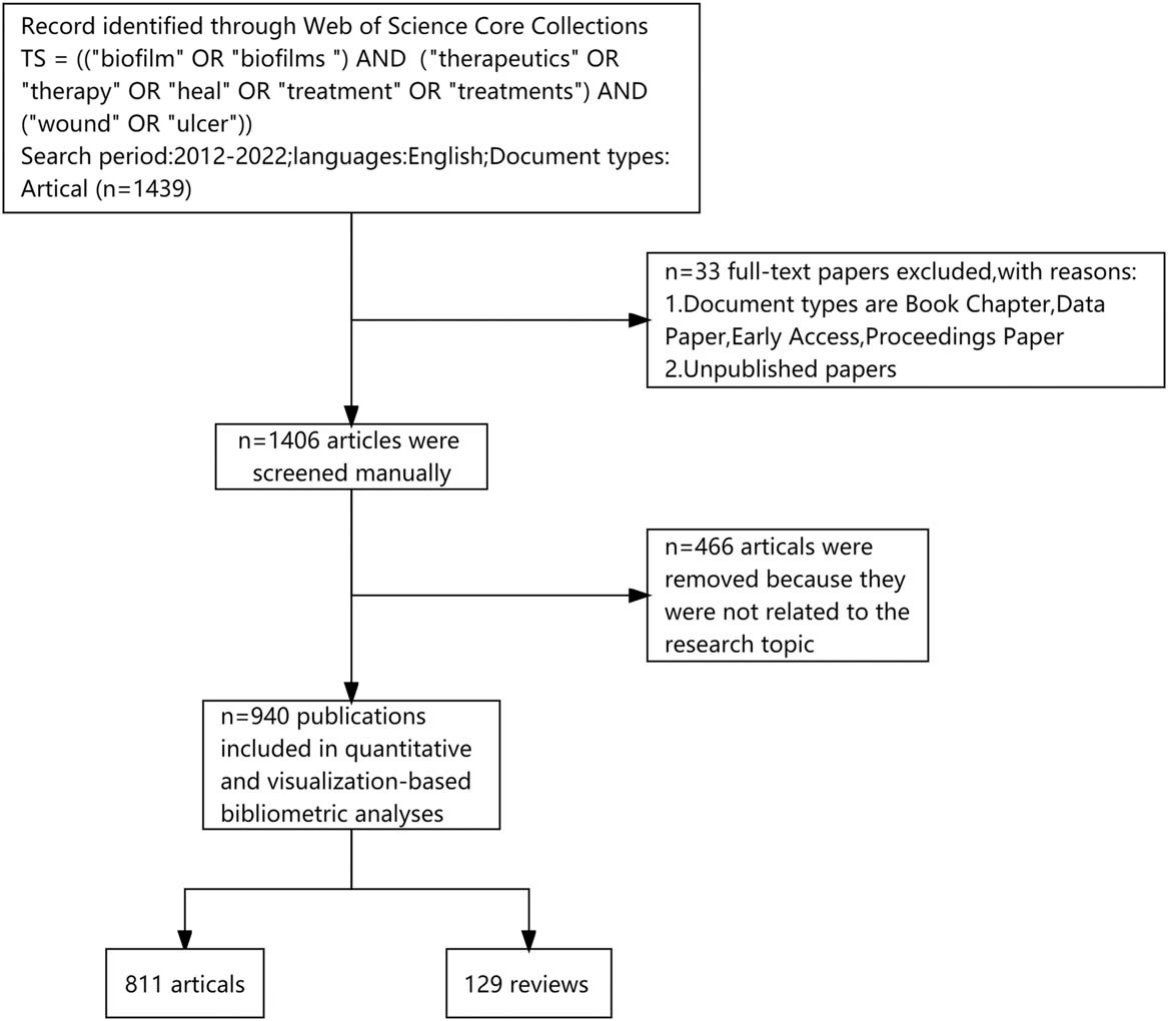
Literature metrology data screening, extraction flow chart.

The core set of Web of Science (WoS) was selected as the database to obtain the initial data due to its groundbreaking content, high-quality data, and long history^8^. Therefore, we searched the Web of Science Core Collection (WoSCC) for papers on bacterial biofilm treatment of wounds from 2012 to 2022. The following keywords were used: TS = ((“biofilm” OR “biofilms”) AND (“therapeutics” OR “therapy” OR “heal” OR “treatment” OR “treatments”) AND (“wound” OR “ulcer”)). Figure 1 shows the specific selection process.

### Data extraction and analysis

The two researchers exported key information from the Web of Science core collection as TXT files, including document source, title, author, country (region), affiliated institution, abstract, keywords, publication year, and reference, and then imported it to Microsoft Excel for data management and analysis. In case of inconsistency, a third, more experienced investigator was invited. Citations and charts for each country or region are analyzed through Microsoft Excel 2021.

CiteSpace 6.1 software was used to conduct literature data cleaning and keyword emergence analysis, which helped summarize the research frontiers of cluster research and reveal the research frontiers of bacterial biofilm wound treatment. Through VOSviewer1.6.18, cluster analysis was conducted on the key information of the literature, such as countries, institutions, journals, authors, keywords, etc., to construct and visualize the symbiotic network of important terms extracted from the literature^9^. In addition, the study assessed journal impact factor (IF) and keyword trend maps from bibliometrics, a bibliometric project launched by Dr. Massimo Aria and Dr. Corrado Cuccurullo in 2017. Comprehensive mapping analysis tools were used in the R software environment^10^.

## Results

### Statistics of publications results

**Figure 2:**
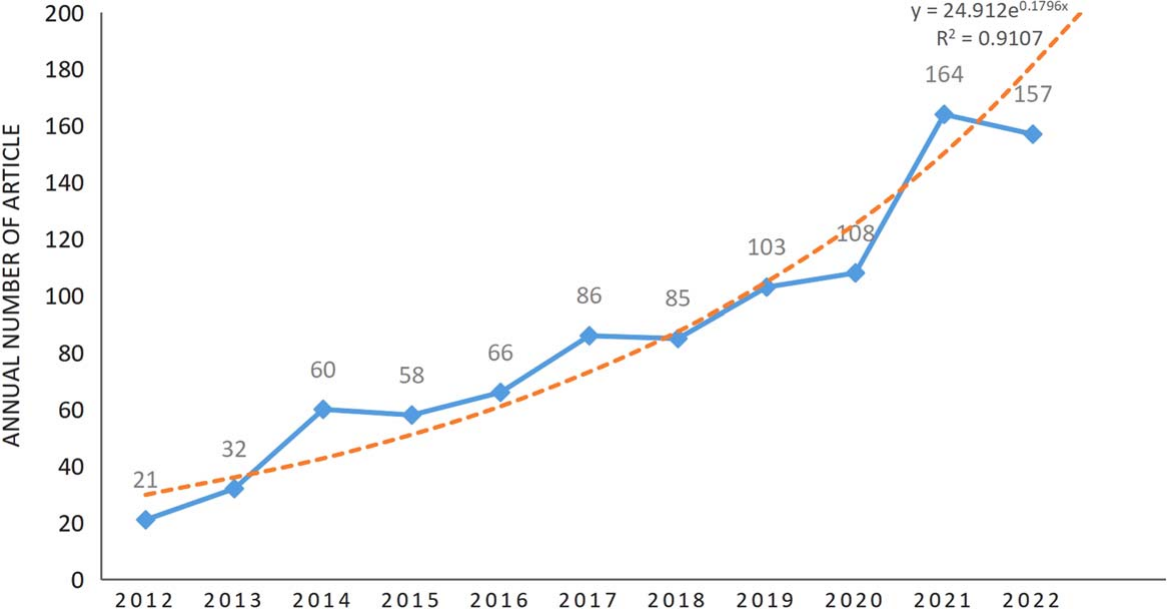
Paper on bacterial biofilm wound treatment from 2012 to 2022.

This study used bibliometrics to analyze the research of bacterial biofilm in wounds, focusing on main countries, institutions, authors, and journals, and to explore the research trends and hotspots. Ultimately, 940 articles met the criteria and were included in the analysis (including 811 articles and 129 reviews). The change in the number of papers generally reflects the change in the development direction of the research field, and is also an important symbol of the research hotspot of the discipline^11^. The number of studies reporting on bacterial biofilm wound treatment increased steadily from 2012 to 2022 and achieved a substantial increase in 2020, reaching 164 publications in 2021, which is about 8 times more than that in 2012 (21) (Fig. 2), indicating a higher need and awareness to study bacterial biofilm-related to wound healing. Although the current research has made great achievements, there are still many uncertainties in the growth mechanism of bacterial biofilm and the efficacy of wound treatment. To better treat bacterial biofilms and promote wound healing, more efforts are needed to study the potential mechanisms of bacterial biofilms and find effective therapeutic targets.

### Journal contributions and research category

The 940 articles included in this study were published in 341 journals, and the top 10 journals in terms of literature publication were from the United States (5), the United Kingdom (3), and Switzerland (2) (Table 1). The top 10 journals accounted for 25% of the total articles published in this study. Journal of Wound Care, with an IF of 2.066, published the largest number of articles (39 articles, 4.1%), followed by Frontiers in Microbiology (31 articles, 3.1%) and International Wound Journal (26 articles, 2.8%). The journal with the most citations was Wound Repair and Regeneration, with a total citation frequency of 1008 and an average citation frequency of 45.82 per paper.

**Table 1:** The top 10 journals in the field of bacterial biofilm wound treatment from 2012 to 2022

Rank	Journal	Country	Year of publication	Publications	Ratio of composition (%)	Total citations	Citations per paper	IF (2022)	JCR
1	*Journal of Wound Care*	England	1992	39	4.1	625	16.03	2.066	Q4
2	*Frontiers in Microbiology*	Switzerland	2010	31	3.3	634	20.45	6.064	Q1
3	*International Wound Journal*	England	2004	26	2.8	631	24.27	3.099	Q3
4	*ACS Applied Materials & Interfaces*	USA	2009	24	2.6	494	20.58	10.383	Q1
5	*Scientific Reports*	England	2011	23	2.4	584	25.39	4.996	Q2
6	*Wound Repair and Regeneration*	USA	1993	22	2.3	1008	45.82	3.401	Q2
7	*Antimicrobial Agents and Chemotherapy*	USA	1972	21	2.2	863	41.10	5.938	Q1
8	*Antibiotics-Basel*	Switzerland	1953	17	1.8	353	20.76	5.222	Q1
9	*Advances in Wound Care*	USA	2012	16	1.7	762	47.63	4.947	Q3
10	*PLoS One*	USA	2006	16	1.7	458	28.63	3.752	Q2

IF, impact factor; JCR, journal citation reports.

**Figure 3:**
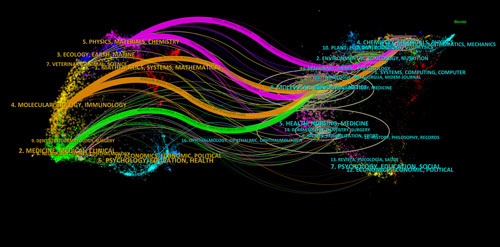
A dual-map overlay of the journals on bacterial biofilm wound therapy research.

The subject distribution which shows the relationship between periodicals is well presented in Citespace software. The citing and cited journals are placed on the left and right sides of Figure 3, respectively, and the cited trajectory and knowledge flow are shown by the colour path diagram^12^. The pink routes show that molecular/biology/genetics and chemistry/materials/physics journal papers were frequently cited by physics/materials/chemistry journals. The two orange lines represent molecular/biology/immunology journals that typically cite articles published in molecular/biology/genetics and chemistry/materials/physics journals. Green Paths prompt articles published in medical/medical/clinical journals that are primarily noted and cited by molecular/biology/genetics journals. It is suggested that the research field of bacterial biofilm wound treatment has attracted the attention of molecular, biology, medicine, physics, materials, and chemistry journals.

### Analysis of National (regional) Contributions and international cooperation

**Figure 4:**
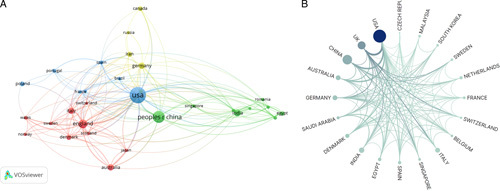
National/regional social network analysis.

A total of 1386 institutions from 80 countries (regions) were included in this study. The top 10 countries with the highest number of publications are listed in Table 2. USA had the most publications and citations and dominated the field of bacterial wound biofilm treatment, with 302 articles (accounting for 32.13%) and 8573 citations, followed by China (166 articles, 2863 citations), the United Kingdom (90 articles, 2966 citations), India (53 articles, 794 citations), and Germany (43 articles, 1462 citations). Cluster analysis showed that 31 countries (regions) had published papers more than 10 times, and these countries and regions were divided into four clusters according to the number of co-authored articles (Fig. 4A). The first green cluster includes China, India, Egypt, Singapore, South Korea, and Saudi Arabia; Group 2 (red) includes the United Kingdom, Italy, Australia, Denmark, Sweden and Japan; Germany, Canada, Iran and Russia are clustered in the third yellow cluster. Group 4 (blue) includes the United States, Poland, Portugal, Spain, France and Brazil. The United States has strong partnerships with China, the United Kingdom and Australia (Fig. 4B).

**Table 2:** Top 10 countries in terms of contributions to bacterial biofilm wound treatment from 2012 to 2022

Rank	Country	Publications	Ratio of composition (%)	Total citations	Citations per paper
1	USA	302	32.13	8573	28.39
2	China	166	17.66	2863	17.25
3	England	90	9.57	2966	32.96
4	India	53	5.64	794	14.98
5	Germany	43	4.57	1462	34
6	Italy	43	4.57	1583	36.81
7	Australia	42	4.47	1636	38.95
8	Denmark	31	3.30	1794	57.87
9	Iran	29	3.09	422	14.55
10	Poland	26	2.77	382	14.69

### Research institution’s contributions

**Figure 5:**
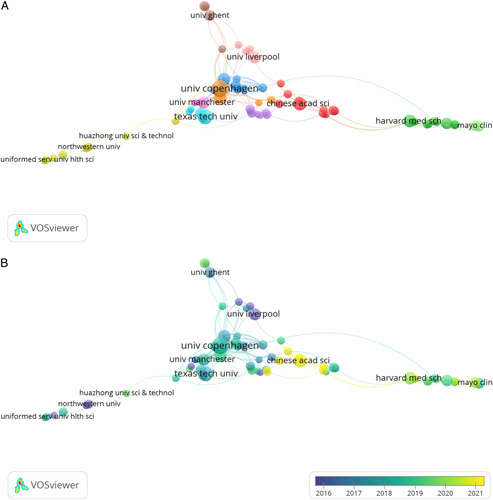
Social network analysis chart (A) and time series analysis chart (B) of research institutions.

Table 3 shows the top 10 institutions with the greatest contribution to the field, where the University of Copenhagen ranked first with 26 articles (2.77%), followed by Texas Tech University (*n*=20, 2.13%), the Chinese Academy of Sciences (*n*=16, 1.70%), Copenhagen University Hospital (*n*=15, 1.60%), and the University of Florida (*n*=15, 1.60%). According to the social network analysis diagram (Fig. 5A), 11 clusters were found, among which the institutions of concern are as follows the University of Copenhagen, Texas Tech University, the Chinese Academy of Sciences, Ghent University, the University of Liverpool, the University of Manchester, Harvard Medical School, Ohio State University, Baylor University of the US Army, Washington State University, and University of Birmingham. The sequence chart (Fig. 5B) indicates that the University of Liverpool in the UK published earlier, more authoritative, and more influential studies on the treatment of bacterial biofilm wounds, while research institutions such as the Chinese Academy of Sciences, although starting late, with higher potential for the future.

**Table 3:** Top 10 institutions in the field of bacterial biofilm wound treatment from 2012 to 2022

Rank	Institution	Country	Publications	Ratio of composition (%)	Total citations	Citations per paper
1	University of Copenhagen	Denmark	26	2.77	1715	65.96
2	Texas Tech University	USA	20	2.13	798	39.90
3	Chinese Academy of Sciences	China	16	1.70	513	32.06
4	Copenhagen University Hospital	USA	15	1.60	472	31.47
5	University of Florida	USA	15	1.60	662	44.13
6	Harvard Medical School	USA	15	1.60	481	32.07
7	Ohio State University	USA	14	1.49	873	62.36
8	University of Liverpool	England	13	1.38	924	71.08
9	University of Manchester	England	13	1.38	281	21.62
10	Tehran University of Medical Sciences	Iran	12	1.28	190	15.83

The cluster analysis of institutions suggests that the Chinese Academy of Sciences, University of Copenhagen, Texas Tech University, University of Liverpool, and Harvard Medical School are the most representative institutions in the field. Therefore, future research on bacterial biofilm therapy of wounds is most likely to come from the above institutions. Also, actively seeking cooperation with these institutions may improve the quality of research on bacterial biofilm therapy of wounds and promote the development of this field.

### Author contributions

A total of 5054 authors participated in the research results in this field; Table 4 lists the 10 most productive authors. Among them, six authors published more than 10 papers, among which Steven L Percival ranked first with 14 papers and 1146 citations, contributing the largest in the field, followed by American scholars Kai P Leung (12 articles), Haluk Beyenal (12 articles), Robin Patel (10 articles), Kendra P Rumbaugh (10 articles), and Danish scholar Thomas Bjarnsholt (10 articles), who ranked first in the number of citations per paper (93.3 citations), which can be focused on his research direction, the role of biofilms in chronic infections. Leung Kai P and his team published 12 articles with 515 citations. Chinese scholars were not included in the top 10. Thus, future cooperation with Chinese and foreign authoritative institutions may improve the research level in this field based on more high-quality research.

**Table 4:** Top 10 authors of English articles on bacterial biofilm wound treatment from 2012 to 2022

Rank	Author	Country	Publications	Ratio of composition (%)	Total citations	Average per year	Co-author	Citations	Total links strength
1	Steven L Percival	England	14	1.49	1146	81.86	Steven L Percival	384	8130
2	Kai P Leung	USA	12	1.28	515	42.92	Randall D Wolcott	361	7591
3	Haluk Beyenal	USA	12	1.28	129	10.75	Thomas Bjarnsholt	294	6699
4	Robin Patel	USA	10	1.06	72	7.20	Costeron J W	271	5394
5	Thomas Bjarnsholt	Denmark	10	1.06	933	93.3	James GA	257	4813
6	Kendra P Rumbaugh	USA	10	1.06	431	43.10	Niels Høiby	172	4000
7	Abdulrahman Mohamed	USA	9	0.96	67	10.78	Stewart P S	186	3508
8	Claus Moser	Denmark	8	0.85	64	8.00	Dowd SE	134	3146
9	Tom Coenye	USA	8	0.85	226	28.25	Luanne Hall-Stoodley	132	2922
10	Robert D Galiano	USA	7	0.74	324	46.29	Sean Donlan	154	2813

### Co-cited literature analysis

**Figure 6:**
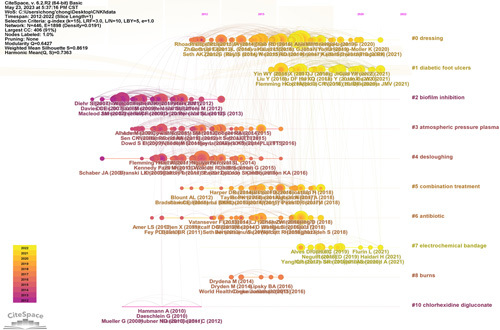
Co-cited references timeline map of publications on the application of bacterial biofilm wound therapy research from 2012 to 2022.

Total citations can be used as an indicator of the impact of an article and the level of attention paid to scientific issues in the field^13^. Among the top 10 most frequently cited articles from 2012 to 2022 (Table 5), two were from *Wound Repair and Regeneration*. The main research fields of the *Wound Repair and Regeneration* journal were the evaluation and treatment of diabetic foot, influencing factors, and molecular biology. The article with the highest number of citations (583 times) was published in the *Journal of Pathology, Microbiology, and Immunology* in 2013; the role of bacterial biofilms in chronic infection in microbiology and immunology emphasizes that bacterial biofilms are usually present in chronic wounds. Supporting the view that biofilm lifestyle dominates bacterial infection in chronic wounds, and assuming the survival mode of bacterial biofilm in chronic wound infection, provides the basis and inspiration for more research, improved diagnosis, treatment, and biofilm treatment^14^. The next most cited paper was ESCMID* Guidelines for the Diagnosis and Treatment of Biofilm Infections 2014,which provides guidelines for diagnosing and treating biofilms^15^. The reference timeline map (Fig. 6) suggested that the current research focuses on the electrochemical bandage. Studies have also shown that low concentrations of H_2_O_2_ and HOCl can be found in wound beds, are generated as part of natural cellular inflammatory responses, and are noteworthy for their inherent potential properties in eliminating biofilms in wound beds and stimulating wound healing. The continuous generation and delivery of H_2_O_2_ and HOCl to wound beds to reduce biofilms could be considered ideal antibacterial effects^16,17^. To combat bacteria biofilms, researchers have taken advantage of this property to develop and evaluate the in-vitro anti-biofilm activity of a novel proof-of-concept electrochemical bandage system (E-bandage) consisting of carbon fabric, a 3-electrode system controlled by a wearable potentiostatic device, which uses xanthan gel-based hydrogels to provide electrolytic conductivity between electrodes, continuously producing controlled amounts of H_2_O_2_ and HOCl^18–20^.

**Table 5:** Top 10 journals with the highest number of English articles on bacterial biofilm wound treatment from 2012 to 2022.

Rank	Year	First author	Title	Source	Total citations	IF (2022) (JCR)
1	2013	Thomas Bjarnsholt	The Role of bacterial biofilms in chronic infections	*Journal of Pathology, Microbiology, and Immunology*	583	Not found
2	2014	Hoiby	ESCMID* guideline for the diagnosis and treatment of biofilm infections 2014	*Clinical Microbiology and Infection*	434	13.310 (Q1)
3	2014	Martina Abrigo	Electrospun Nanofibers as Dressings for Chronic Wound Care: Advances, Challenges, and Future Prospects	*Macromolecular Bioscience*	345	5.859 (Q3)
4	2012	Steven L, Percival	A review of the scientific evidence for biofilms in wounds	*Wound Repair and Regeneration*	302	3.401 (Q3)
5	2014	Lawrence R. Mulcahy	Pseudomonas aeruginosa Biofilms in Disease	*Microbial Ecology*	259	4.192 (Q2)
6	2018	Chen Zhao Wei	Enzyme Mimicry for Combating Bacteria and Biofilms	*Accounts of Chemical Research*	226	24.466 (Q1)
7	2014	Stephanie DeLeon	Synergistic Interactions of Pseudomonas aeruginosa and Staphylococcus aureus in an In Vitro Wound Model	*Infection and Immunity*	207	3.609 (Q2)
8	2014	Steven L, Percival	The effects of pH on wound healing, biofilms, and antimicrobial efficacy	*Wound Repair and Regeneration*	187	3.401 (Q3)
9	2018	Anja Pfalzgraff	Antimicrobial Peptides and Their Therapeutic Potential for Bacterial Skin Infections and Wounds	Frontiers in Pharmacology	186	5.988 (Q2)
10	2014	Michael Zakrewsky	Ionic liquids as a class of materials for transdermal delivery and pathogen neutralization	*Applied Biological Sciences*	181	12.799 (Q1)

IF, impact factor; JCR, journal citation reports.

### Analysis of hotspots in bacterial biofilm wound treatment

**Figure 7:**
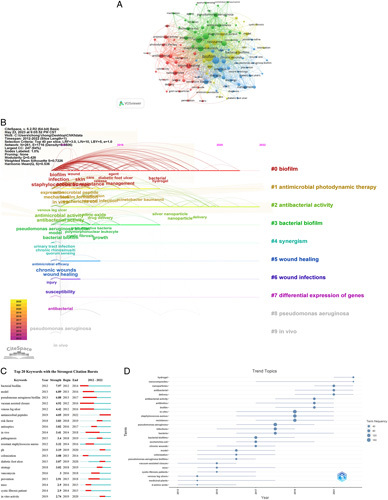
Social network analysis diagram (A), time series analysis diagram (B), emergence diagram (C), and trend diagram (D) of keywords in bacterial biofilm wound treatment.

VOSviewer was used to extract keywords from the abstract titles of 940 studies, generating 115 keywords that appeared at least 13 times. These keywords are grouped into four clusters (Fig. 7A), which include clinical-related research on promoting wound healing-specific therapy, in-vitro experiments of bacterial biofilm, and mechanism research on inhibiting biofilm. The first cluster (blue) includes biofilms, infections, bacteria, treatment, effects, management, and debridement. The second cluster (red) includes wound healing, antibacterial, nanoparticles, antibacterial activity, silver nanoparticles, release, drug delivery, and photodynamic therapy. The third cluster (green) includes in-vitro data on *Pseudomonas aeruginosa*, *Staphylococcus aureus (S. aureus)*, infections, antibiotics, *Escherichia coli*, antimicrobial resistance, biofilm formation, and toxicity. The fourth cluster (yellow) includes biofilms, inhibition, chronic wounds, gold and glucose, mechanisms, skin, models, and sensitivity. Figure 7B shows a plot of the timeline for 10 clusters of research hotspots. Over time, the core hotspots of the cluster continue to develop more research directions (#0 biofilm, #2 antibacterial activity). Among them are nanoparticle, delivery, and gold nanoparticles which reflect the latest research trends.

According to the keyword emergence and trend chart (Fig. 7C, D), the research in the field of bacterial biofilm wound treatment has gradually transferred from the drug resistance of common bacterial biofilms Staphylococcus aureus and Pseudomonas aeruginosa in wounds, venous leg ulcers, and vacuum-assisted healing to the research of chronic wound biofilms, in-vitro bacterial biological models, growth factors and other therapies, and then to more microscopic directions such as inhibition of bacterial activity, drug delivery, and nanomaterials. We believe that nanotechnology, as a wound dressing and drug delivery, is a core field of current attention. Meanwhile, the target group of bacterial biofilm wound treatment is gradually expanding to chronic wounds with a single disease, indicating that bacterial biofilm treatment of chronic wounds has gradually become a difficulty in current medicine and a future research hotspot.

## Discussion

### Research trends in wound bacterial biofilm therapy

Bacterial biofilms seriously affect wound healing, cause serious economic burdens and loss of life and property of patients, and seriously affect the prognosis of patients^21^. Therefore, in-depth research is urgently needed. By evaluating the total number of citations in a country (region), it is possible to measure the academic influence of this country (region) in a specific field^22^. Denmark ranked eighth in the number of articles published, but its average citation frequency ranked the first among all countries (31, 57.84), indicating that Danish and American research institutions made a huge contribution to the field. China ranked second and third among all countries and regions in terms of the number of articles published and the total number of citations, but the average number of citations only ranked 10^th^ among the top 10 countries and regions, suggesting that China should seek cooperation with international authorities to improve the quality of clinical research. Although the United States has so far led the way in this area, China, as the largest developing country, has a large number of patients with wound infections. With the increase in China’s national strength and its strong investment in scientific research, research platforms, and capabilities are gradually improving, and it is believed that multidisciplinary cooperation will produce more and more high-quality articles in China^13^. Based on this, the quantity and quality of domestic research on bacterial biofilm wound treatment will continue to grow, and its research strength and influence have been seriously underestimated.

*Frontiers in Microbiology, ACS Applied Materials & Interfaces, Antimicrobial Agents* and *Chemotherapy* suggest that the medical field is not the only one that pays attention to bacterial biofilm management, as Microbiology, antibiotic research and development, biological materials, and other disciplines are also interested in this field, proving that bacterial biofilm wound treatment is currently research hotspot. It should be noted that the quality of literature related to clinical studies in the top 10 journals with the number of published papers is relatively low, which may be because standardized diagnosis and treatment measures for wound bacterial biofilms have not yet been formed, and high-quality clinical multi-centre randomized controlled trials cannot provide conclusive evidence, so it is necessary to optimize research design and improve research quality in order to standardize the diagnosis and treatment of bacterial biofilms in infected wounds.

The University of Copenhagen in Denmark has contributed the most in this field, with 26 publications; these articles have mainly reported on the mechanism of bacterial biofilm growth. Texas Tech University, which ranked second in the number of publications, focused more on preventing biofilm formation on medical devices and biofilm dispersion research. The Chinese Academy of Sciences has focused on novel materials for the prevention and inhibition of bacterial biofilms, indicating that the research on bacterial biofilms of wounds by the Chinese Academy of Sciences has a certain international influence, mainly because of the scientific research strength of the Chinese Academy of Sciences, the demand for wound healing and the strong investment and support for scientific research. Moreover, the University of Liverpool ranked first in the number of citations, suggesting that attention be paid to the direction of the institution’s research in this field.

Steven L Percival from the United Kingdom is a leader in this field, focusing on clinical research, Including Biofilms, Wounds, Infection Prevention and Control, Medical Devices, and Antimicrobials, and has made great contributions to the research of wound bacterial biofilm treatment, suggesting that focus on its research direction and hotspots.

### The research focus is wound bacterial biofilm therapy

The research focuses on the clinical research of bacterial biofilm treatment of wounds, the basic research of biofilm growth mechanisms, and the development of new materials. According to the Recommendations for the Management of Biofilm: a consensus document^23^, it is pointed out that at present, there is no standardized unified standard for bacterial biofilm treatment of wounds.

The most cited literature was Thomas Bjarnsholt’s doctoral thesis, which recognized the role of bacterial biofilm in chronic infection (Table 5) and explained the biofilm growth mechanism, diagnosis, treatment, and prevention measures^14^. Early detection is essential to prevent biofilm formation at the wound site in the management strategy of wound bacterial biofilms. Traditional biofilm detection techniques include microbiological, DNA-based and RNA-based, and microscopic imaging assays^6^. The biofilm on the wound bed can detect specific diagnostic biomarkers, which can be used as an indicator to determine the status of the wound^24^. More and more studies have focused on sensors due to their demonstrated potential to accurately detect different wound biofilm-related markers and provide reliable information about wound status, including specific bacterial species and wound biofilm EPS sensors, sensors indicating environmental parameters, such as pH and temperature, and enzyme sensors^6^. These research directions may show great potential in the specific diagnosis of wound infection in the future.

*Staphylococcus aureus* and *Pseudomonas aeruginosa* are the leading inhibitors of chronic wound healing^25^, the strong toxicity of its pathogens and strong biofilm resistance, which seriously hinder wound healing, have led to extensive research (Fig. 7A, D), to build a wound biological model, study the biofilm growth mechanism, and test the inhibition ability of new materials on biofilm.

The current standard treatment procedure for wound biofilms consists of frequent removal of biofilms, including physical and chemical debridement and topical and systemic application of antimicrobial agents and dressings^5,26^. Bacterial infection caused by biofilms has become an increasingly serious global health problem, while avoiding bacterial resistance, it is critical to develop alternative antimicrobial regimens^27^. Wound debridement can be classified into surgical, autolytic, biological (involving maggots), mechanical, and enzymatic (collagenase), among others^28^. Doctors need to evaluate individual patient needs and available resources, and select appropriate debridement techniques to achieve the best clinical results^29^. In addition, wound bacterial biofilms cannot be resolved by debridement alone^30^, and a combination of therapeutic cleansing and topical antibacterial therapy is often required^31^. Clinically, sulfamethoxazole, penicillin, streptomycin, tetracycline, vancomycin and other antibiotics are commonly used to treat wound infection, but widespread clinical use may contribute to global antibiotic resistance crisis^32^. Antiseptics such as Octenidine hydrochloride, polyhexamethylene biguanide, povidone-iodine, and Sodium hypochlorite, due to its high bactericidal and anti-biofilm forming activities, has a good indication for wound infection and can be used as a substitute for antibiotic resistance for local treatment^2^. Potential future treatments for infected wounds include antimicrobials (antibiotics, herbal medicines, and synthetics), immune-based antimicrobial molecules (antimicrobial peptides), therapeutic microorganisms (probiotics and bacteriophages), and cell therapy. External stimuli such as antimicrobial phototherapy (NIR-based therapies), laser therapy, light-emitting diode, high-frequency ultrasound, and microcurrent electrical stimulation have also been developed in this field^33^. At the same time, it has been found that an appropriate drug delivery system is particularly important to avoid bacterial resistance and cytotoxicity of preservatives. In this context, special attention needs to be paid to antimicrobial delivery systems, ranging from semi-solid and liquid formulated wound dressings to more advanced drug carriers of nanoparticles, vesicles, electrospun fibres, and micronicles, to investigate their potential to effectively treat wound infections^31^. The ideal wound dressing should help the wound form a physical defense barrier against bacteria, but allow oxygen exchange^34^. Many new wound dressings, including collagen, sponges, hydrogels, hydro-fibres, hydrocolloids, fucoidan, and films, have been widely used in clinical wound treatment, among which antibacterial wound dressings can be divided into antibacterial agents, ion/nanocrystalline silver and antibiotics^33^. In particular, the attention to hydrogels has shifted from traditional wound dressing to thermal, Ph-sensitive, and photosensitive intelligent hydrogels wound dressing^35^. Meanwhile, combination therapy may be more beneficial for infected wounds. For example, impregnated local negative pressure and silver foam have been shown to work collaboratively to destroy bacterial biofilms in wounds^36^. In addition, in mouse models, silver nanoparticles and neomycin showed a strong synergistic effect on the multidrug-resistant bacterium Pseudomonas aeruginosa, accelerating wound healing^37^.

Keyword trend topics(Fig. 7D) suggest that we need to focus on the impact of nanotechnology and drug delivery on wound healing. Work in nanotechnology is well underway to help improve health by developing novel materials, drug and delivery systems, and biomacromolecule separation, and also for the development of new methods, materials, and devices for wound biofilm diagnosis and treatment, which will improve the selection of clinical solutions for proper and effective management of the disease^38^.

Drug delivery systems including surface coatings, fibres, and nanoparticles intelligently target the site of infection, effectively enhance the bacteria-killing effect, and reduce potential side effects on normal tissue, and the formation of bacterial biofilms^39^. However, the substrate of the carrier, the interaction between the drug and the carrier, and the physical and chemical properties of the drug may affect the release rate of the drug delivery system^40^. At the same time, future antimicrobial applications urgently need more requirements, such as intelligence, self-regulated release, and targeted drug delivery^41^.

## Conclusion

This study suggests that the article reporting on bacterial biofilm wound therapy has been gradually increasing year by year. Yet, most studies are *in vitro* and animal trials, while clinical research accounts for a small number of articles. It is predicted that future studies may combine in-vitro biofilm model with clinical practice to actively promote the clinical use of nanoparticles, hydrogels, chitosan, and other new biological materials and drugs, to obtain a large sample randomized controlled trial of best practice evidence and explore more efficient and safe intervention methods to remove wound bacterial biofilm.

Of course, there were some limitations. First, this study only included the Web of Science, which may lose some high-quality literature and lead to inaccurate conclusions. At the same time, the field of wound bacterial biofilm was not deeply studied in this study. In the future, it is necessary to extract more bacterial biofilm wound treatments included in professional databases at home and abroad and obtain more authoritative bibliometric conclusions. Furthermore, in the study of wound biofilm management strategies, it is necessary to fully account for the synergistic effect of multidisciplinary nursing combined treatment, consider the individual differences of different wound sites, formulate more accurate personalized treatment plans, emphasize the importance of specialized wound care, summarize the intervention process of wound bacterial biofilm, and accelerate wound healing.

## Ethical approval

This review does not require ethical approval.

## Consent

Informed consent is not required for this review.

## Sources of funding

Mechanism research and clinical application of bacterial biofilm microenvironment in the wound(YF-HZ-20230101).

## Author contribution

R.L. and L.Z. were responsible for the conception of research ideas, paper writing, and data analysis; S.F. and R.G. were responsible for data collection and collation; Supervisor J.Z. was responsible for the revision and approval of the final version.

## Conflicts of interest disclosure

The authors declare that we have no conflict of interest.

## Guarantor

Jie Zheng.

## Data availability statement

The data in this study are publicly available.

## Provenance and peer review

Not commissioned, externally peer-reviewed.
